# Mozart K.545 Mimics Mozart K.448 in Reducing Epileptiform Discharges in Epileptic Children

**DOI:** 10.1155/2012/607517

**Published:** 2012-12-05

**Authors:** Lung-Chang Lin, Mei-Wen Lee, Ruey-Chang Wei, Hin-Kiu Mok, Hui-Chuan Wu, Chin-Lin Tsai, Rei-Cheng Yang

**Affiliations:** ^1^Department of Pediatrics, School of Medicine, College of Medicine, Kaohsiung Medical University, Kaohsiung City 807, Taiwan; ^2^Department of Pediatrics, Kaohsiung Medical University Hospital, Kaohsiung Medical University, Kaohsiung City 807, Taiwan; ^3^Department of Music, National Sun Yat-Sen University, Kaohsiung City 804, Taiwan; ^4^Institute of Applied Physics and Underseas Technology, National Sun Yat-Sen University, Kaohsiung City 804, Taiwan; ^5^Institute of Marine Biology, National Sun Yat-Sen University, Kaohsiung City 804, Taiwan; ^6^Department of Neurology, Kaohsiung Municipal Hsiao-Kang Hospital, Kaohsiung Medical University, Kaohsiung City 807, Taiwan

## Abstract

Mozart K.448 has been shown to improve cognitive function, leading to what is known as the Mozart Effect. Our previous work reveals positive effects of Mozart K.448 in reducing epileptiform discharges in epileptic children. In this study, we evaluated the effect of Mozart K.545 and compared the effects with those of Mozart K.448 on epileptiform discharges in children with epilepsy. Thirty-nine epileptic children with epileptiform discharges were included in the study. They received electroencephalogram examinations before, during, and after listening to Mozart K.448 and K.545, one week apart, respectively. The frequencies of epileptiform discharges were compared. There was a significant decrease in the frequency of epileptiform discharges during and right after listening to Mozart K.448 and K.545 (reduced by 35.7 ± 32.7% during Mozart K.448 and 30.3 ± 44.4% after Mozart K.448; and 34.0 ± 39.5% during Mozart K.545 and 31.8 ± 39.2% after Mozart K.545). Spectrogrammatic analysis of the two pieces of music demonstrated that both share similar spectrogrammatic characteristics. Listening to Mozart K.448 and K.545 decreased the epileptiform discharges in epileptic children. This suggests that Mozart K.448 is not the only piece of music to have beneficial effects on children with epilepsy. Other music with lower harmonics may also decrease epileptiform discharges in epileptic children.

## 1. Introduction 

Music has been used to improve physical and mental illnesses. Rauscher et al. first report the “Mozart Effect” in 1993. They note that Stanford-Binet spatial task scores improve immediately after listening to Mozart's Sonata for Two Pianos in D major, K.448 (Mozart K.448) for ten minutes, when compared to the same time of silence or relaxation instruction [1]. Rauscher suggests that cognitive processing is improved by listening to Mozart's music. Subsequent studies demonstrate the beneficial effects of listening to music for many neurologic diseases, including Parkinson's disease, senile dementia, and sleep disorder [2–4]. Regarding epilepsy, Hughes et al. and our previous study show that the epileptiform discharges decrease when listening to Mozart K.448 in patients with epilepsy [5, 6]. In addition, our study shows that harmonics are associated with decreasing epileptiform discharges. However, whether Mozart K.448 is the only piece of music that can effectively reduce epileptiform discharges remains unclear. In the present study, we used another piece of Mozart's music, Mozart Piano Sonata No. 16 in C major (Mozart K.545), with similar harmonics to Mozart K.448, to study the role of the harmonics of the musical stimulus in reducing epileptiform discharges. We analyzed the relationships between the decrease in epileptiform discharges with the foci of epileptiform discharges, mentality, state of wakefulness, epileptic etiology, seizure type, and gender. 

## 2. Patients and Methods

### 2.1. Subjects

Thirty-nine Taiwanese children (19 boys and 20 girls) diagnosed with epilepsy were enrolled. The mean age of these children was 7 years 3 months ±3 years 5 months (ranging from 2 years 9 months to 17 years 3 month). The diagnosis of epilepsy was made according to the criteria established by the International League Against Epilepsy (ILAE). Informed consent was given by a family member or legal guardian in each case. This study was approved by the Institutional Review Board of Kaohsiung Medical University Hospital. 

### 2.2. Electroencephalogram Examinations

The patients in this study received electroencephalogram (EEG) examinations with three sections of parallel periods; before, during, and after listening to Mozart K.448 (8 min 22 sec) and K.545 (9 min 7 sec) in random order, one week apart, respectively. They received 60–70 dB of musical stimuli via loudspeakers [7] that was measured with a decibel meter (DSL332, Taipei, Taiwan). Each EEG was recorded digitally (Harmonie DVN V5.1, Montreal, Canada). Electrodes were placed according to the International 10–20 System. Two neurologists counted the number of discharges in each of the three sections of the experiment. Changes in epileptiform discharge were expressed as (baseline discharge − discharge during/after music/baseline discharge) ×100. Each patient maintained the same state of wakefulness throughout the recording period. We defined an “effective” result as exposure to the music resulting in a reduction of epileptiform discharges by more than 20% (about half the value of one standard deviation of decreased epileptiform discharges in this study).

### 2.3. Spectrogrammatic Analysis of Mozart K.448 and K.545

Spectrogrammatic analyses of Mozart K.448 and K.545 were performed with the MATLAB program (Mathworks, Inc., MI, USA). Short-time Fourier transformations of the time signals were computed to generate the time series of spectra (spectrogram). A hamming window was used to truncate 100 s of time data, which was sampled at a rate of 44.1 kHz for each spectrogram. The frequency resolution for the analyzed 20 kHz frequency range was 1 Hz.

### 2.4. Statistical Analysis

Data are shown as means ± SD. Differences in the distribution of effective and noneffective results were calculated using the chi-square test. The two-sample *t*-test and ANOVA were used to compare the percentages of epileptiform discharge reduction while listening to the music by mentality, seizure type, epileptic etiology, state of wakefulness, and gender. Paired *t*-tests were used to compare epileptiform discharge frequencies before, during, and after listening to the music. Pearson correlation coefficients were used to test the correlations of the effects between the two pieces of music. A *P* value less than 0.05 was considered statistically significant.

## 3. Results

Thirty-nine patients with epilepsy were recruited for this study (19 males and 20 females). Thirty-two patients demonstrated normal intelligence, five patients had a reduced IQ, and two patients had undetermined IQ levels. The majority of patients (*n* = 29) were idiopathic in etiology, two patients were probably symptomatic, and eight patients were symptomatic (Table 1). None of the patients were suffering from musicogenic epilepsy or active seizures at the time of study.

### 3.1. Change of Epileptiform Discharge during and after Music Exposure

**Figure 1:**
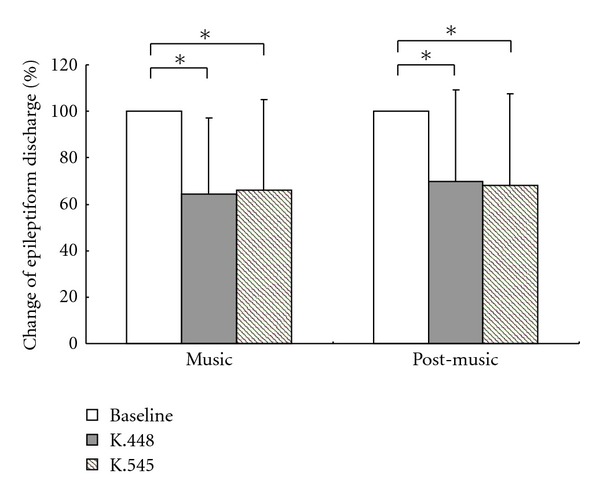
Epileptiform discharges during and after listening to Mozart K.448 and K.545. Comparisons made with baseline EEG (before music). Percentages of the decrease observed in epileptiform discharges in all patients during (*n* = 39) and after listening to Mozart K.448 (*n* = 33) and after listening to K.545 (*n* = 34). *P < 0.001.

Since the average epileptiform discharge frequency in each patient before music listening was highly variable, ranged from 1.1/min to 88.7/min (average 20.9 ± 22.5/min), and 0.4/min to 85.2/min (average 16.9 ± 22.7/min) before Mozart K.448 and Mozart K.545, respectively. The changes of epileptiform discharges were expressed as percentage of reduction. There was a significant decrease in the frequency of epileptiform discharges during listening to Mozart K.448 and K.545 (35.7 ± 32.7% reduction during Mozart K.448 and 34.0 ± 39.5% reduction during Mozart K.545). Thirty-three and thirty-four patients maintained the same stage of sleep or wakefulness during EEG examinations after the conclusion of Mozart K.448 and K.545, respectively. After excluding data from the six and five patients who changed their wakefulness state, the reductions of epileptiform discharges were significant right after listening to Mozart K.448 and K.545 (30.3 ± 44.4% a reduction after Mozart K.448 and 31.8 ± 39.2% after Mozart K.545) (Figure 1). Most patients (84.6% and 82.1% during Mozart K.448 and K.545, resp.) demonstrated decreased interictal discharge frequencies when listening to either piece of music. The patients with generalized epilepsy had more effective results than those with focal seizures when listening to Mozart K.448 and K.545 (*P* = 0.048 and 0.024, resp.) (Table 1). However, six and seven patients had an increase in interictal discharge frequencies in the Mozart K.448 and K.545 music group, respectively. Most of them (66.7% and 71.4%) had occipital discharges. There was no significant difference in the change of epileptiform discharges between patients who were awake and asleep during the EEG recordings. In addition, there were no significant differences in epileptiform discharges by gender, epilepsy etiology, and IQ (Table 1).

**Figure 2:**
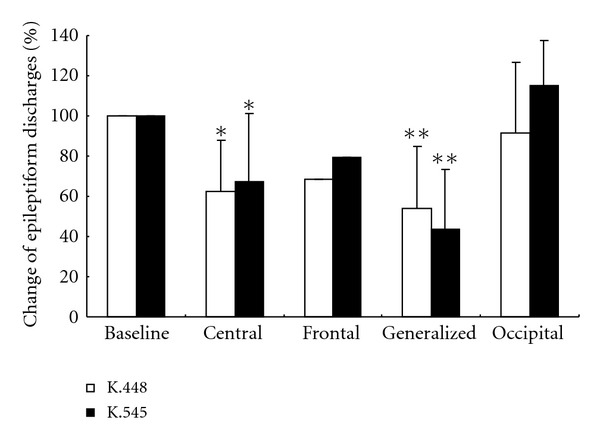
Epileptiform discharge reduction by area of epileptic focus. Comparisons made with baseline EEG (before music) baseline, central (*n* = 11), frontal (*n* = 1), generalized (*n* = 19) and occipital (*n* = 8). *P < 0.01, ^∗∗^ *P* < 0.001.

When the epileptiform discharge foci in patients were considered, eleven showed central origin, one had frontal origin, nineteen demonstrated generalized discharges, and eight had occipital origin. The average reductions of epileptiform discharges in different foci when listening to Mozart K.448 and K.545 were 37.6 ± 25.2% and 32.7 ± 34.1% from central origin; 31.4%, and 20.4% from the frontal origin; 46.2 ± 31.1% and 56.1 ± 29.3% from generalized discharges; and 8.8 ± 35.7% and −15.2 ± 22.3% from occipital origin, respectively. Compared with the baseline data before music stimulation, the reductions were significant in patients with central and generalized discharges for both pieces of music (Figure 2). 

### 3.2. Spectrograms and Correlations of the Reduction in Epileptiform Discharges between Mozart K.448 and Mozart K.545

**Figure 3:**
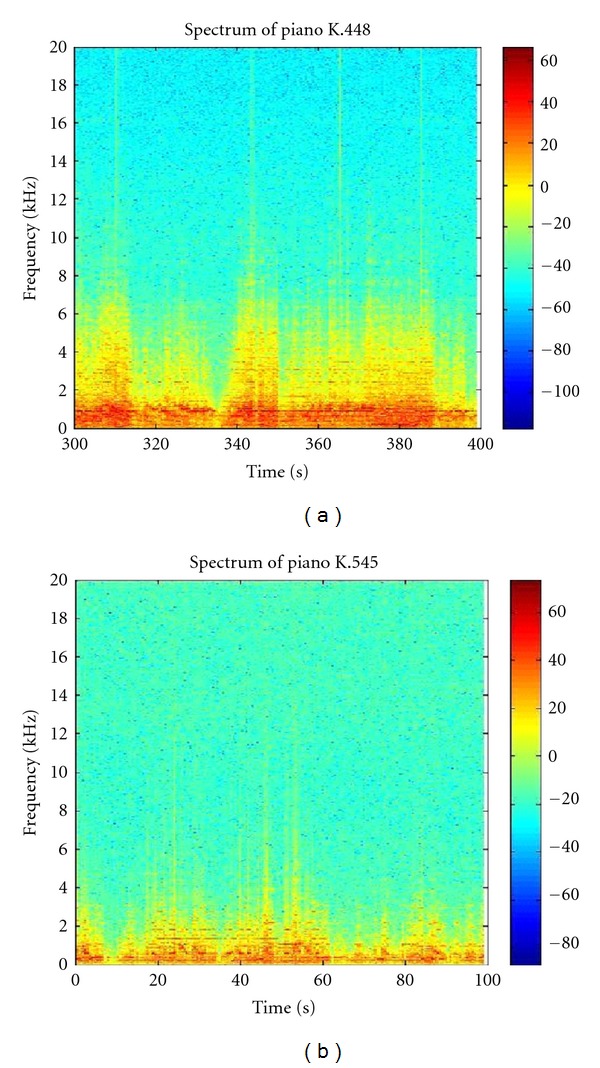
Spectrogram analysis for Mozart K.448 and K.545. The spectrograms showed similar low frequency harmonics during one section of Mozart K.448 (a) and K.545 (b). Data were averaged in 5-second periods in the middle (300 s) of the first movement of K.448, and initial (0 s) of the first movement of K.545.

**Figure 4:**
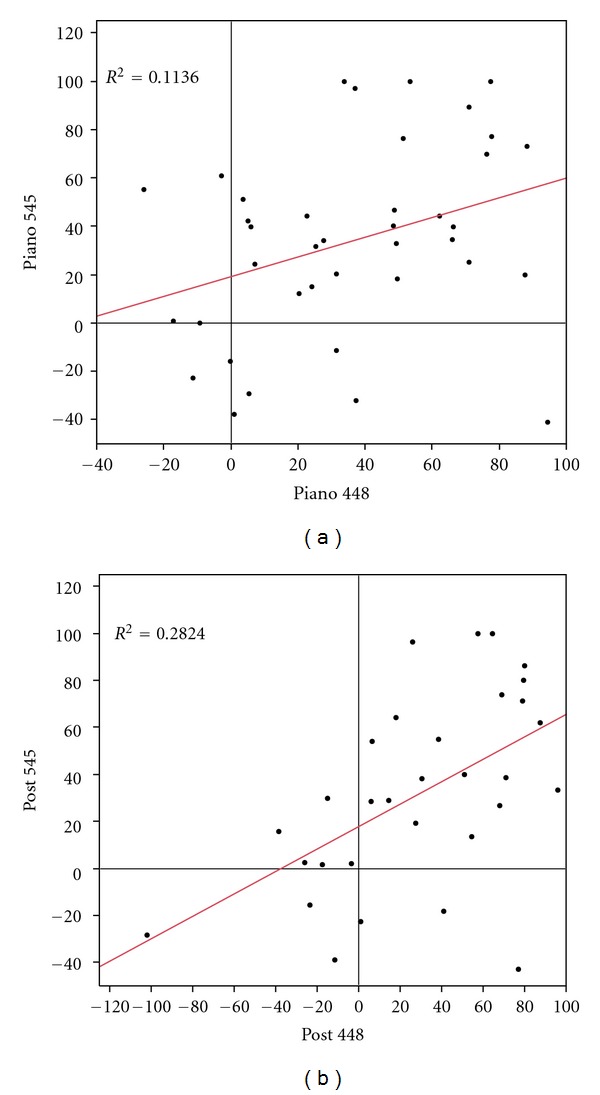
Correlation of reduction of epileptiform discharges between Mozart K.448 and K.545. There was a significant correlation in epileptiform discharge reduction between Mozart K.448 and K.545 during (a) and right after (b) listening to the music.

Although Mozart K.448 has a more complex music structure and time variation than K.545, both still share similar spectrogrammatic characteristics (Figures 3(a) and 3(b)). The similarities are particularly noteworthy at lower frequencies, where both concentrate more energy in the fundamental frequencies and the lower harmonics, and both peak at around 1 kHz. Significant correlations in decreased epileptiform discharges were observed between Mozart K.448 and K.545 during (correlation coefficient = 0.337, *P* = 0.036) and right after (correlation coefficient = 0.531, *P* = 0.002) listening to the music (Figures 4(a) and 4(b)).

## 4. Discussion

There are a number of recent studies reporting the positive effects of music in patients with neurological diseases. Patients with migraines receive 12 weeks of music-aided relaxation training and demonstrate a significant reduction in headache frequencies, suggesting a possible mechanism of relaxation [8]. Patients with middle cerebral artery stroke show a significant improvement in recovery of verbal memory and focused attention after listening to their favorite music for two months [9]. It is postulated that the enhanced cognitive recovery is possibly the result of positive emotions induced by the music. Another study shows that short-term, self-selected music improves aim and line tracking in patients with Parkinson's disease [10]. Our previous studies show that listening to Mozart K.448 reduces epileptiform discharges as well as seizure frequencies [6, 11]. It is clear, therefore, that music has positive effects on several neurological diseases.

Musical characteristics including rhythm, melody, texture, form, tone color, and tonality may play roles in the beneficial effects of music. Hughes reports that the long-term periodicity of the melody line in Mozart's music has a significantly higher value when compared with music by Wagner and three other composers. Hughes suggests this may be the reason why Mozart's music reduces epileptiform discharges [12]. Zhao and Chen report that the valence of music melodies, rather than the mood it induces, may be the mediator in the pain-relief effect of different pieces of music [13]. Our previous study shows that listening to string K.448 with higher harmonics does not decrease epileptiform discharges when compared with the patients who listen to piano K.448 [6]. In our current study, we hypothesized that the similar lower harmonics in Mozart K.448 and K.545 may be the crucial factor responsible for the reduction in epileptiform discharges. In this study, we confirmed that both Mozart K.448 and K.545 showed significant correlations in the reduction of epileptiform discharges.

Although the human auditory cortex is found in the temporal area, listening to music is a complex process for the brain, since it triggers a sequence of cognitive and emotional responses [14]. Neural activity associated with listening to music extends beyond the auditory cortex. It involves a wide-spread bilateral network of frontal, temporal, parietal, and subcortical areas associated with attention, semantic and music-syntactic processing, memory, motor functions, even extending to the limbic and paralimbic regions related to emotional processing [15–23]. Since our results demonstrated that the greatest decrease in epileptiform discharges occurred in patients with generalized and central discharges, the results are clearly not limited to an effect in the auditory cortex. 

In this study, most patients who had increase in epileptiform discharges during music stimulation demonstrated the epileptic foci of occipital origin. The reason patients with occipital foci did not have significant decreases or even increases in epileptiform discharges after music listening remains unclear. On the basis of the evidence from mirror neuron studies, when musical stimuli enter the temporal cortex, areas of the ventral premotor cortex and areas of the inferior parietal lobule are activated and considered to form a frontoparietal mirror neuron system [24]. The results from this study suggested that the occipital cortex did not appear to be involved in this auditory network.

There were no significant differences in gender, epileptic etiology, and state of wakefulness in this study, which we also report in our previous study [6, 25] and is noted in other studies. In addition, Dureau reports no significant gender differences in heart rate and behavioral state after listening to music for 3 minutes [26]. Significant differences were not found when analyzing IQ levels in our study. Research indicates that music therapy is a useful therapeutic approach, regardless of mentality, and is commonly used with mentally retarded children or adults. Our previous study shows that IQ levels were not associated with the reduction of epileptiform discharges in epileptic children when listening to Mozart K.448 [6]. In children with Rett syndrome, active music therapy improves fine motor tasks and social behavior [27], and in a study by Heal and O'Hara, handicapped women with Down's syndrome show improvement in their relationship to the external world and in anorectic behavior after music therapy [28].

Recently, several theories have been introduced regarding the effects of sound on the brain. It is reported that poor health is associated with lower parasympathetic tone in several medical conditions, including epilepsy [29]. Mukherjee et al. report that lower parasympathetic tone, lower parasympathetic reactivity, and more severe dysautonomia are found in patients with intractable epilepsy than in those with well-controlled epilepsy [30]. One study shows that a two-hour music intervention in cancer patients increases their relaxation scores and parasympathetic activities [31]. Another study shows that forty-five minutes of music therapy once per week in patients with cerebrovascular disease enhances parasympathetic activities and decreases congestive heart failure events by reducing plasma cytokine and catecholamine levels [32]. It is possible that musical enhancement of parasympathetic tone may account for the beneficial effects on epilepsy. 

Neurotransmitter pathways may also be involved in the effect of Mozart K.448 and K.545 on epilepsy. Musical exposure is known to increase the expression of dopamine levels in the brain [33]. In recent years, dopamine is reported to play a crucial role in the pathophysiology of epilepsy. The reduced binding capacity of dopamine receptors in the basal ganglion is hypothesized to contribute to seizures in autosomal dominant frontal lobe epilepsy and juvenile myoclonic epilepsy [34]. In a recent animal study, the authors report that pilocarpine induces seizures by altering the affinity of dopaminergic receptors in striatal and hippocampal areas, facilitating the propagation and maintenance of seizures [35]. It is possible that listening to music modifies the dopaminergic pathways contributing to the beneficial effects in epilepsy therapy. 

## 5. Conclusions

In conclusion, listening to Mozart K.448 and K.545 decreased the epileptiform discharges in children with epilepsy. The effect was most pronounced when the discharges originated from the central cortex or were generalized. Our findings suggest that Mozart K.448 is not the only piece of music to have beneficial effects on children with epilepsy, and that listening to Mozart K.545 with similar lower harmonics can decrease epileptiform discharges in epileptic children as well. 

## Figures and Tables

**Table 1:** Profile comparison between patients effective and noneffective in decrease epileptiform discharges during Mozart K.448 and K.545 exposure.

	K.448	K.448	*P* value	K.545	K.545	*P* value
	Effective	Noneffective		Effective	Noneffective	
Sex						
Male (%)	13 (68.4)	6 (31.6)	0.915	15 (79)	4 (21)	0.113
Female (%)	14 (70)	6 (30)		11 (55)	9 (45)	
Mentality						
IQ ≧ 70 (%)	23 (71.9)	9 (28.1)	0.722	22 (68.8)	10 (31.2)	0.813
IQ < 70 (%)	3 (60)	2 (40)		3 (60)	2 (40)	
Undetermined (%)	1 (50)	1 (50)		1 (50)	1 (50)	
Seizure type						
Generalized (%)	16 (84.2)	3 (15.8)	0.048	16 (84.2)	3 (15.8)	0.024
Focal (%)	11 (55)	9 (45)		10 (50)	10 (50)	
Classification						
Idiopathic (%)	21 (72.4)	8 (27.6)	0. 092	20 (69)	9 (31)	0.826
Probably symptomatic (%)	0 (0)	2 (100)		1 (50)	1 (50)	
Symptomatic (%)	6 (75)	2 (25)		5 (62.5)	3 (37.5)	
Conscious state						
Awake (%)	17 (70.8)	7 (29.2)	0.784	16 (66.7)	8 (33.3)	1.000
Sleep (%)	10 (66.7)	5 (33.3)		10 (66.7)	5 (33.3)	
